# Cattleianal and Cattleianone: Two New Meroterpenoids from *Psidium cattleianum* Leaves and Their Selective Antiproliferative Action against Human Carcinoma Cells

**DOI:** 10.3390/molecules26102891

**Published:** 2021-05-13

**Authors:** Engy A. Mahrous, Ahmed M. Al-Abd, Maha M. Salama, Magda M. Fathy, Fathy M. Soliman, Fatema R. Saber

**Affiliations:** 1 Pharmacognosy Department, Faculty of Pharmacy, Cairo University, Kasr el-Aini Street, Cairo 11562, Egypt; engy.abdelhamid@pharma.cu.edu.eg (E.A.M.); maha.salama@pharma.cu.edu.eg (M.M.S.); drmagda@elkhlawiw.com (M.M.F.); fathy.soliman@pharma.cu.edu.eg (F.M.S.); fatema.saber@pharma.cu.edu.eg (F.R.S.); 2 Department of Pharmaceutical Sciences, College of Pharmacy & Thumbay Research Institute of Precision Medicine, Gulf Medical University, Ajman 4084, United Arab Emirates; 3 Pharmacology Department, Medical Division, National Research Centre, Cairo 11562, Egypt; 4 Pharmacognosy Department, Faculty of Pharmacy, The British University in Egypt, El-Sherouk City, Cairo 11837, Egypt

**Keywords:** meroterpenoids, phloroglucinol, *Psidium cattleianum*, myrtaceae, cytotoxicity, cell cycle, apoptosis/necrosis

## Abstract

The Myrteacae family is known as a rich source of phloroglucinols, a group of secondary metabolites with notable biological activities. Leaves of *Psidium cattleianum* were extracted with chloroform: methanol 8:2 to target the isolation of phloroglucinol derivatives. Isolated compounds were characterized using different spectroscopic methods: nuclear magnetic resonance (NMR), ultra-violet (UV) and mass spectrometry (MS). Two new phloroglucinols were evaluated for cytotoxicity against a panel of six human cancer cell lines, namely colorectal adenocarcinoma cells (HT-29 and HCT-116); hepatocellular carcinoma cells (HepG-2); laryngeal carcinoma (Hep-2); breast adenocarcinoma cells (MCF7 and MDA-MB231), in addition to normal human melanocytes HFB-4. Additionally, cell cycle analysis and annexin-V/FITC-staining were used to gain insights into the mechanism of action of the isolated compounds. The new phloroglucinol meroterpenoids, designated cattleianal and cattleianone, showed selective antiproliferative action against HT-29 cells with IC_50_’s of 35.2 and 32.1 μM, respectively. Results obtained using cell cycle analysis and annexin-V/FITC-staining implicated both necrosis and apoptosis pathways in the selective cytotoxicity of cattleianal and cattleianone. Our findings suggest that both compounds are selective antiproliferative agents and support further mechanistic studies for phloroglucinol meroterpenoids as scaffolds for developing new selective chemotherapeutic agents.

## 1. Introduction

Myrtaceous plants exert a myriad of biological activities including anti-diabetic, cytotoxic, anti-inflammatory and anti-bacterial biofilm activities [1,2,3]. *Psidium cattleianum* Sabine (Family Myrtaceae) is a shrub or small tree native to Brazil, where it is known as “araçá” [4]. It is now cultivated throughout the tropics and subtropics for its juicy purple-red fruits know as strawberry guava or Cattley guava [5]. In a recent report, ninety-three compounds were identified in the aqueous leaves′ extract of *P. cattleianum* where catechin, gallic acid and vanillic acid were reported as the major constituents [6].

Leaves of *P. cattleianum* are used in folk medicine as an anti-hemorrhagic, antispasmodic and anti-diarrheal agent [7], but recent investigations have uncovered a selective anti-proliferative effect of *P. cattleianum* leaf extract against human carcinoma cells [8,9]. Additionally, a closely related species, *Psidium guavaja,* showed cytotoxic activity in prostate cancer cell lines that was mediated through the induction of apoptosis [10]. Phytochemical investigation of *P. guajava* resulted in the isolation of sixteen phloroglucinol meroterpenoids which all displayed cytotoxicity against five human cell lines with IC_50_ reported below 60 μM [11]. Among isolated meroterpenoids, psiguajavadial A and psiguajavadial B induced apoptosis in human cancer cell line HCT116 in the nano-molar range. Most recently, six novel phloroglucinol meroterpenoids were isolated from *Psidium littorale* Raddi which all showed cytotoxic activity against several human cancer cell lines [12,13] emphasizing the potential of phloroglucinol meroterpenoids as versatile and selective cytotoxic agents. Therefore, this study was designed to target the isolation of novel phloroglucinols from *P. cattleianum* and investigate their possible anti-proliferative activity.

## 2. Results

### 2.1. Identification of the Isolated Compounds

Two new meroterpenoids were isolated from the chloroform: methanol extract of *Psidium cattleianum* leaf. Structure elucidation of the isolated compounds was carried out based on their *m/z*, recorded using high-resolution mass spectrometry HR-MS, UV absorbance and 1D and 2D-NMR spectra.

Compound **1** was isolated as yellow oil, soluble in chloroform, Rf = 0.56 in S1, ${\left[\alpha \right]}_{D}^{25}$-75 (c 0.4, CHCl_3_). It showed a yellow color under a UV lamp (λ_max_, 365 nm). EI-MS identified pseudomolecular ion [M-H]^−^ at *m/z* = 397 for C_24_H_29_O_5_ with fragment ions appearing at *m/z* 195 (95%), 161 (47%) and 203(28%), (Supplementary Data, Figure S1). ^1^H-NMR *δ* ppm (400 MHz, CDCl_3_):*δ_H_* 0.93 (3H, s, H-12), 0.97 (3H, s, H-13), 1.38 (1H, m, H-1), 1.37 (1H, m, H-3a), 1.47 (3H, s, H-14), 1.49 (2H, m, H-2), 1.65 (1H, m, H6a), 1.72 (2H, m, H-10), 1.74 (2H, m, H5), 1.76 (1H, m,H-7a), 1.77 (1H, m, H-6b), 2.05 (1H, m, H-3b), 2.23 (1H, m, H-7b), 2.4 (2H, m, H-9), 2.88. (1H, m, H-9″a), 3.04 (1H, m, H-9′b), 4.84 (1H, br s, H-15a), 4.86 (1H, br s, H-15b), 9.92 (1H, s, H7′), 10.11 (1H, s, H-8′), 12.96 (1H, s,4′ OH), 13.22 (1H, s, 6′-OH), (Supplementary Data, Figure S2). ^13^C-NMR *δ* ppm (100 MHz, CDCl_3_):*δ_C_* 20.7 (C-13), 22.4 (C-14), 23.7 (C-10), 24.1 (C-2), 27.9 (C-9′), 28.6 (C-12), 34.4 (C-11), 34.9 (C-7), 39.5 (C-6), 40.7 (C-5), 41.9 (C-9), 46.9 (C-3), 58.3 (C-1), 87.02 (C-4), 103.1 (C-3′), 104.6 (C-5′), 104.7 (C-1′), 110.1 (C-15), 149.8 (C-8), 162.9 (C-2′), 166.8 (C-4′), 166.9 (C-6′), 191.04 (C-7′), 191.06 (C-8′), (Supplementary Data, Figure S3).

Based on NMR spectra, the presence of a fully substituted phloroglucinol ring was inferred by the carbon signals (C-1′-C-6′), while no aromatic proton was observed in the ^1^H-NMR spectrum. Two phenolic substituents characteristic of phloroglucinol were readily determined in ^1^H-NMR at *δ_H_* 12.96 (s, 1H) and 13.22 (s, 1H), which showed multiple HMBC correlations to aromatic carbons at δ_C_ 166.8, 166.9, 103.1 and 104.7, (Supplementary Data, Figure S4). Additionally, the presence of 3,5 formyl substitutions was established by the two carbon signals (C-7′ and C-8′) and aldehydic proton signals at *δ_H_* 9.92 (s, 1H) and 10.11 (s, 1H) which showed HMBC correlations to C-1′, C-3′, C-4′ and C-6′ (Supplementary Data, Figure S4), establishing the 3,5 formyl phloroglucinol moiety characteristic for *Psidium* species [13,14,15]. This 3,5 formyl phloroglucinol moiety produced the characteristic EI-MS fragment ion at *m/z* 195 (95%).

**Figure 1:**
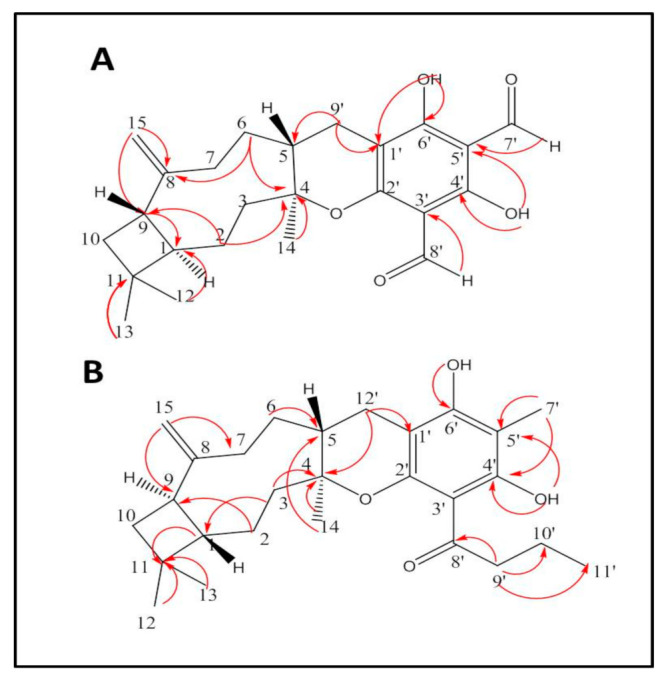
Structure and key HMBC correlations for (**A**): Cattleianal and (**B**): Cattleianone.

Another EI-MS fragment at *m/z* 203 was consistent with the sesquiterpene moiety of β-caryophyllene as previously reported for littordial A-E, guajadial A and pisidial A [12,16,17]. ^13^C-NMR and ^1^H-^13^C-HSQC established the caryophyllene moiety encompassing three methyls, six methylenes, three methine and three quaternary carbons. The presence of a pyran ring connecting the phloroglucinol and sesquiterpene moiety was inferred from quaternary carbon (C-4) at *δ_C_* 87.02 and its HMBC correlation to the phloroglucinol (H-9′ 2H, 2.88, 3.04), which in turn showed clear HMBC correction to the aromatic carbon C-1′ at *δ_C_* 104.7 ppm, Figure 1, supplementary material Figures S4–S7. To complete the assignment of the sesquiterpene moiety, an exocylic double bond was evidenced by the presence of methylene group (C-15), observed in the HSQC spectrum at δ_H/C_ 4.84,4.86/110.1, Figures S5 and S6. Also, a cyclobutane ring was determined by the characteristic chemical shift for C-1 at *δ_H/C_* (1.38, m/58.3), characteristic of other phloroglucinol meroterpenoids psidial A and littordial B and C [11,12,13,14]. Further analysis of HMBC and HSQC spectra of compound **1** and careful comparison of the observed chemical shifts of C-1, C-3 and C-6 with that of the chemical literature of other phloroglucinols suggested that compound **1** is most structurally related to littordial B and C which were previously isolated from *P. littorale* [12] and pisidial A [11], previously isolated from *P. guajava* which all share 1R,4R,5S,9S stereochemistry, as can be inferred from the chemical shifts of C-1, C-3 and C-6 [15,18]. Accordingly, compound **1** was identified as a β-caryophyllene-phloroglucinol derivative and, based on the presence of two aldehyde groups, was designated as cattleianal, Figure 1.

Compound **2** was isolated as dark yellow oil, soluble in chloroform, ${\left[\alpha \right]}_{D}^{25}$ + 85.7 (c 0.7, CHCl_3_). High resolution ESI-MS in negative mode showed a pseudo molecular ion [M-H]^−^ at *m/z* 425.26865 calculated for C_27_H_37_O_4_ and [2M-H]^−^ ion at *m/z* 851.54559, (Supplementary Data, Figure S8). UV spectrum of compound **2** (MeOH) showed UV maximum at 294 nm, (Supplementary Data, Figure S9). ^1^H-NMR δ ppm (400 MHz, CDCl3):*δ_H_* 0.89 (3H, t, J = 6.7 Hz, H-11′), 0.94 (3H, s, H-12), 1.14 (3H, s, H-14), 1.29 (1H, m, H-2a), 1.47 (1H, m, H-6a), 1.59 (2H, m, H-10), 1.62 (1H, m, H-6b), 1.73 (2H, m, H-10′), 1.83 (1H, m, H-2b), 1.87 (1H, m, H-3a), 1.93 (1H, m, H-1), 1.99 (3H, s, H-7′), 2.05 (1H, m, H-12′a), 2.08 (2H, m, H-5), 2.13 (1H, m, H-3b), 2.16 (1H, m, H-7a), 2.38 (1H, s, C-9), 2.4 (1H, m, H-7b), 2.54 (1H, m, H-12′b), 2.99 (1H, m, H-9′a), 3.1 (1H, m, H-9′b), 4.78 (1H, br s, H-15a), 4.81 (1H, br s, H-15b), 14.09 (1H, s, 4′OH), 14.1 (1H, s, 6′OH), Supplementary Figure S10. ^13^C-NMR δ ppm (100 MHz, CDCl3):δ_C_ 6.02 (C-7′), 12.9 (C-11′), 17.5 (C-10′), 21.1 (C-14), 21.6 (C-12), 22.05 (C-2), 25.07 (C-12′), 29.3 (C-13), 33.7 (C-5), 34.4 (C-6), 34.8 (C-11), 35.2 (C-7), 35.5 (C-10), 36.9 (C-3), 41.1 (C-9), 45.5 (C-9′), 51.7 (C-1), 81.06 (C-4), 98.7 (C1′), 100.4 (C-5′), 108.8 (C-3′), 109.1 (C-15), 151.2 (C-8), 153.6 (C-2′), 156.4 (C-6′), 161.5 (C-4′), 205.1 (C-8′). The ^1^H-NMR spectrum of compound **2** was similar to that of cattleianal except for the absence of the two aldehyde protons indicating lack of formyl substituents, (Supplementary Data, Figure S10). Another significant difference was the appearance of two methyl groups at *δ_H_* 1.99 (s, 3H) and 0.89 (t, J = 6.7, 3H). ^13^C-NMR showed the characteristic signals of β-caryophyllene moiety and confirmed lack of formyl substitution, (Supplementary Data, Figure S11). Instead, ^13^C-spectrum showed a signal for a quaternary carbon at 205.1 ppm consistent with an aromatic ketone and an upfield methyl group at *δ_C_* 6.02 with clear HMBC correlations to *δ_C_* 161.5, 156.4, Supplementary Data, Figures S12 and S13. This indicated the position of the methyl substituent (C-7′) between two aromatic carbons with hydroxyl groups C-4′ and C-6′, respectively. Also, the unusual upfield chemical shift in the aromatic methyl can be explained by its *m*-position to the ketone substituent at *δ_C_* 205.1.

Meanwhile, the carbonyl of the ketone group was traced via HMBC correlation to a C3 aliphatic chain (C-9′-C-11′), in addition to the correlations observed in the COSY spectrum (Figures S16 and S17), establishing the presence of 3′-butanoyl, 5′-methyl-benzene moiety instead of 3′, 5′-diformyl benzyl commonly seen in *Psidium* meroterpenoids [14,15]. To the best of our knowledge, littrodial D is the only meroterpenoid with β-caryophyllene moiety and ketone substituent of the phloroglucinols identified so far [12,18]. However, no derivative has been isolated in which the other formyl group is replaced with a methyl substituent as observed in compound **2**. Accordingly, compound **2** was designated as cattleianone, a new β-caryophyllene meroterpenoid with 3′-butanoyl, 5′-methylphloroglucinol.

### 2.2. Cytotoxicity Assessment of Cattleianal and Cattleianone

Cytotoxicity evaluation of cattleianal and cattleianone against a panel of human cell lines indicated that both compounds have weak cytotoxic activity against HCT-116 cells with calculated IC_50_’s above 70 μM. However, HepG2 and HT-29 were more susceptible to the antiproliferative/cytotoxic effects of both compounds, Table 1. This highlights the selectivity of both compounds, in which IC_50_’s could not be determined against normal human melanocytes (HFB4 cells) up to concentration > 100 μg/mL.

### 2.3. Influence of Cattleianal and Cattleianone on Cell Cycle Distribution

**Figure 2:**
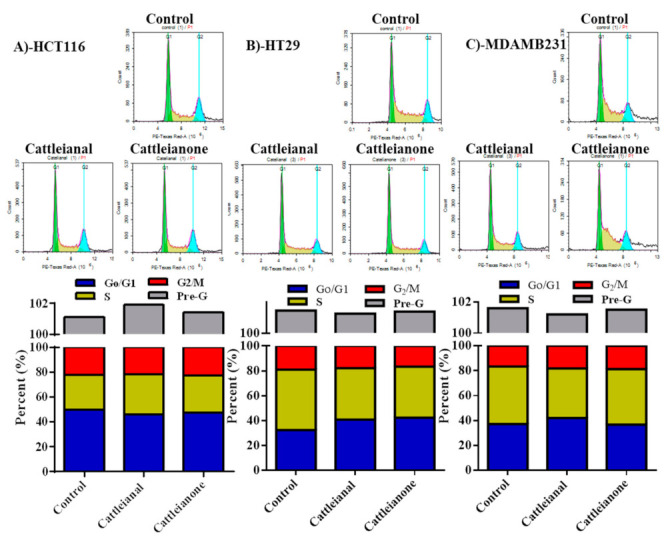
Cell cycle analysis after 24 h incubation with Cattleianal and Cattleianone at their IC_50._ Cell cycle analysis using flow cytometry and diagrammatic presentation of the results after the incubation of Cattleianal and Cattleianone with HCT-116 (**A**), HT-29 (**B**) and MDA-MB-231 (**C**) at their IC_50_’s.

Neither cattleianal nor cattleianone induced any significant change in cell cycle distribution of HCT-116 cells after 24 h incubation (Figure 2A), while both compounds exerted a significant anti-proliferative effect in HT-29 cells with increased cell population in G0/G1-phase from 32.4 ± 0.6% to more than 40.8 ± 2.1% (Figure 2B). Reciprocally, cattleianal and cattleianone significantly decreased proliferative S-phase cells from 48.6 ± 0.8% to 41.4 ± 1.6% and 41.4 ± 3.9%, respectively. In addition, cattleianone significantly decreased cells in G2/M-phase from 19.0 ± 0.3% to 16.5 ± 0.1% (Figure 2B).

Similarly, but to a lesser extent, only cattleianal exerted anti-proliferative effects against MDA-MB-231 cells and increased the population of cells in G0/G1-phase from 37.1 ± 1.6% to 41.9 ± 1.1% with reciprocal decrease in the S-phase population from 46.2 ± 1.9% to 39.9 ± 1.4% (Figure 2C).

### 2.4. Apoptosis/Necrosis Assessment Using Flow Cytometry

**Figure 3:**
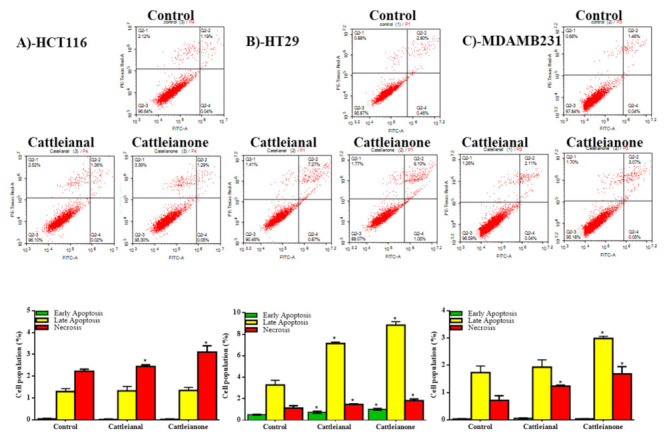
Apoptosis/necrosis assessment of cell population after 24 h incubation with Cattleianal and Cattleianone at their IC_50_’s. Apoptosis/necrosis assessment and diagrammatic presentation of the results after 24h incubation of cattleianal and cattleianone with HCT-116 (**A**), HT-29 (**B**) and MDA-MB-231 (**C**) at their IC_50_. * significantly different from control *p* < 0.05.

Plant-derived natural products can often induce cell death by either apoptosis or necrosis, while some, such as resveratrol, are reported to display both pathways [19]. We assessed cell fractions going through apoptosis/necrosis after incubation with both drugs and observed that both compounds induced only necrotic non-programmed cell death in HCT-116 cells, with no significant change in early or late apoptotic cell fractions after 24 h incubation (Figure 3A).

Meanwhile, in HT-29 cells, cattleianal and cattleianone, at their IC_50_ concentration, induced a significant increase in the number of cells undergoing apoptosis (early and late) as well as cells undergoing non-specific necrosis compared to untreated cells (Figure 3B). In MDA-MB-231, only cattleianone induced significant apoptosis while both compounds induced non-programmed cell death (necrosis) when compared to control cells as shown in Figure 3C.

### 2.5. Molecular Assessment of Apoptosis

**Figure 4:**
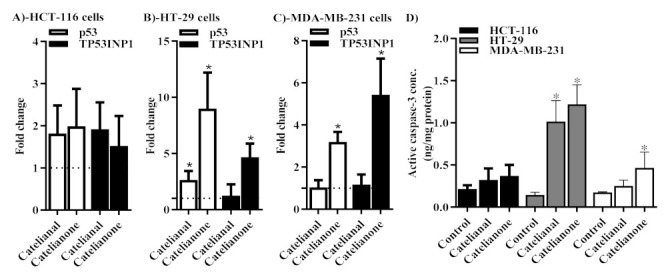
Molecular assessment of apoptosis in after 24 h incubation with Cattleianal and Cattleianone at their IC_50_’s. The expression of *p53* and TP53INP1 genes using qPCR were assessed in HCT-116 (**A**), HT-29 (**B**) and MDA-MB-231 (**C**) cells after 24 h incubation with the IC_50_’s of cattleianal or cattleianone. The active caspase-3 concentration was assessed in HCT-116, HT-29 and MDA-MB cells using ELISA technique and expressed as ng/mg protein (**D**). Dotted line (…) in A, B and C panels represent control gene expression equal to one-fold change. * significantly different from control *p* < 0.05.

Further molecular confirmation of apoptosis was undertaken by assessing the expression level of several key apoptosis related genes using qPCR technique. Both cattleianal and cattleianone induced significant overexpression of the *p53* gene by 2.6 and 9 fold in HT-29 and MDA-MB-231 cells, respectively (Figure 4B,C). This is indicative of apoptosis inducing effects in both compounds under investigation in HT-29 and MDA-MB-231 cells. Only cattleianone induced overexpression of the *p53* phosphorylating gene, TP53INP1 in both HT-29 and MDA-MB-231 cells by 4.6 and 5.4 fold, respectively (Figure 4B,C). This is not only indicative of *p53* overexpressing the effect of cattleianal and cattleianone, but also indicative of *p53* activation effect of cattleianone in these two cell lines. None of the compounds under investigation induced any significant change in the expression of either *p53* or TP53INP1 genes in HCT-116 cells (Figure 4A).

In addition, the concentration of the effective apoptosis executional marker, active caspase-3, was determined after treatment with cattleianal and cattleianone using the ELISA technique. Caspase-3 concentration was significantly higher in response to treatment with cattleianal and cattleianone in HT-29 cells. However, only cattleianone induced a significant increase in the active caspase-3 concentration in MDA-MB-231 cells (Figure 4D).

## 3. Discussion

Phloroglucinols are a class of phenolic secondary metabolites with wide distribution in the Myrtaceae family, but were also reported in other plant families as well as in microorganisms and marine sources [18]. Phloroglucinols can occur as monomers, dimers or as terpene adduct such as phloroglucinol meroterpenoids of *Eucalyptus* and *Psidium* [18]. Phloroglucinols of both genera have been reported to possess cytotoxic effects including euglobals and macrocarpals reported in *Eucalyptus* species and psidials, littrodials from *Psidium* species [11,12,13,20]. Herein, we report the isolation and cytotoxic activity of two new phloroglucinol meroterpenoids, namely cattleianal and cattleianone, from the leaves of *P. cattelianum*. Both compounds are phloroglucinol-β-caryophyllene adducts similar to those reported in other *Psidium* species [13,14]. Both cattleianal and cattleianone lack substituent at C-9′ which set them apart from phloroglucinols of *P. guajava* (often substituted with a phenyl ring at C-9′) and phloroglucinols from *P. littorale*, where C-9′ position is often substituted with C3 and C4 aliphatic chain [13,14]. Preliminary investigations have indicated that several phloroglucinols meroterpenoids displayed cytotoxic activity, including some at the nanomolar range [13,14,15].

Previous reports have established the cytotoxicity of *P. cattleianum* leaves’ extract [10,11], and our findings here point to contributions of cattleianal and cattleianone to this reported cytotoxicity. Cattleianal and cattleianone showed somewhat selective but weak to moderate activity against the investigated cancer cell lines where the cytotoxic activity was more pronounced in Hep-2 and HT-29 cell lines. Interestingly, previous investigation of the cytotoxic potential of *P. cattleianum* leaf extract found similar differential toxicity against cancer cell lines, with human gastric carcinoma (SNU-16) reported as a highly susceptible cell line [8].

The results of cell cycle distribution analysis of cattleianal and cattleianone on HCT-116, HT29 and MDA-MB231 cell lines suggested that the antiproliferative effect of both compounds at their IC_50_ is likely mediated through G_0_/G_1_ cycle arrest. In a previous study, other phloroglucinol derivatives; namely sideroxylonal-B and Macrocarpal-A, showed a similar anti-proliferating effect against human breast carcinoma cells (MCF7), by reducing percentage of cells in the S-phase through increasing percentage of cells in G_0_/G_1_ phase [20].

Further analysis using Annexin V-FITC/PI flow cytometry indicated that both compounds induced early and late apoptosis in HT-29 cells, implicating apoptosis related proteins. Supporting our results, Caspases and PARP proteins were previously reported to be involved in the cytotoxicity of *P. cattelianum* extract against gastric carcinoma cells [8]. Additionally, natural and semisynthetic phloroglucinols have been shown to activate Caspases 3 and 9 with subsequent cleavage of PARP protein in human glioma and breast carcinoma cell lines [16,17]. Therefore, it is likely that Caspases are one of the target proteins mediating the antiproliferative action of cattleianal and cattleianone. Yet, to further examine this hypothesis, *p53* and TP53INP1 gene expression were assessed and found to be induced by cattleianal and cattleianone treatment. The gene *p53* carries a central role in apoptosis and the TP53INP1 gene is responsible for phosphorylation and activation of *p53*-induced apoptosis signaling [21,22]. The elevated concentration of the active form of caspase-3 in response to cattleianal and cattleianone treatment confirms the propagation of apoptosis signal to the late apoptotic phase in affected cell lines [23]

Meanwhile, HCT-116 cells, which were more resistant to both compounds, underwent necrosis showed no significant cell population in early or late apoptosis. A similar pattern was observed for MDA-MB231 breast carcinoma cells, which were more resistant to cattleianal and went through necrosis after incubation with the drug (IC_50_ 64.4 μM), while upon incubation with cattleianone (IC_50_ 38.7 μM), MDA-MB231 cells underwent apoptosis. Thus, these results implicate both programmed and non-programmed cell death in the cytotoxicity effect of cattleianal and cattleianone. It can be argued that the differential activity of both compounds against different cell lines is likely related to the expression levels of their apoptosis–effector targets in each cell line where lower availability of the target protein(s) makes the cells more resistant to the effect of the drugs, and necrosis pathway is triggered as a result. Further investigations into the specific target(s) are therefore recommended for a better understanding of the chemotherapeutic benefits for both drugs.

## 4. Materials and Methods

### 4.1. General Experimental Procedures

Mass spectra were obtained using Varian Mat. 711, Finningan SSQ 7000 and OMM 7070 E. ^1^H, and ^13^C-NMR analyses were recorded on a Bruker AVIIIHD400 FT–NMR Spectrometer (400/3) instrument (Japan). Optical activity was estimated with Kruss polarimeter. Silica gel G 60, reversed phase-C18 silica and silica gel H (E-Merck, Darmstadt, Germany) and Sephadex LH-20 were used for chromatographic procedures.

Thin-layer chromatography (TLC) was performed on silica gel GF254 precoated plates (Fluka, Steinheim, Germany). Solvent systems were: S1: n-hexane: ethyl acetate (95:5 *v*/*v*), S2: n-hexane: ethyl acetate (85:15 *v*/*v*). Doxorubicin^®^ and Sulforhodamine-B were purchased from Sigma-Aldrich (St. Louis, MO, USA). Trichloroacetic acid (TCA) and other materials were of the highest available commercial grade.

### 4.2. Plant Material

*P. cattleianum* was cultivated and collected from the Experimental Station of Medicinal Plants, Faculty of Pharmacy, Cairo University. Samples were kindly identified by Dr. Mohamed El-Gebaly (Senior Botanist). A voucher specimen (PC-12-2018) was kept in the herbarium of Department of Pharmacognosy, Faculty of Pharmacy, Cairo University. Leaves were air dried in shade, powdered and stored in air tight containers.

### 4.3. Extraction and Isolation

One kilogram of powdered leaves was extracted with chloroform: methanol 80:20 in a Soxhlet apparatus following the method described by Sidana et al. [24] for the targeted extraction of phloroglucinols, to yield 114 g of dark residue after solvent evaporation. The extract (62 g) was chromatographed on a VLC column (10 × 15 cm) of silica gel H (330 g). Elution sequence started with n-hexane (100%) followed by 5% increments of EtOAc, up to 100% EtOAc, then CHCl_3_/MeOH (5% increments), up to 20% MeOH. Fractions (200 mL each) were collected and monitored by TLC and fractions with similar TLC patterns were pooled together to yield 4 collective major fractions (Fr. I–Fr. IV).

Fr. I (3 g) showed two major spots, Rf = 0.57, 0.47 in S1 and was chromatographed on a silica gel 60 column (27 × 2.5 cm) eluted with a gradient of 5–10% EtOAc in n-hexane. Forty fractions (10 mL each) were collected and monitored by TLC. Subfraction (16–18) was further purified on a silica gel column (40–63 µm) using 5% EtOAc in n-hexane, fractions (5 mL each) were collected and pooled according to their TLC behavior. Further purification was carried out on an RP-18 silica column using 100% methanol to yield 100 mg of yellow oil (compound **1**).

Subfraction (30–39) was rechromatographed on a sephadex LH-20 column (27 × 1.5 cm) using chroform:methanol (3:1), and fractions (12–25) were collected and purified on successive RP-18 silica columns using methanol:water (1:1) and then 100% methanol and finally on a Sephadex LH-20 column (100% methanol). This afforded 120 mg of dark yellow oil (compound **2**).

### 4.4. Cell Culture

Human breast adenocarcinoma cells (MCF-7 and MDA-MB-231), and colorectal adenocarcinoma cells (HT-29 and HCT-116), were obtained from Nawah Scientific (Cairo, Egypt). Human laryngeal carcinoma (HEp2), hepatocellular carcinoma (HepG2) and human normal melanocytes (HFB4) were obtained from the National Cancer Institute, Kasr El Aini, Cairo, Egypt. MCF-7 and MDA-MB-231 cells were maintained in DMEM media while other cell lines were maintained in RPMI-1640 media. All media were supplemented with 100 µg/mL streptomycin, 100 units/mL penicillin and 10% heat-inactivated fetal bovine serum. Cells were passaged in a humidified incubator at 37 °C with 5% (*v*/*v*) CO_2_ atmosphere.

### 4.5. Cytotoxicity Assays

Cytotoxicity was tested by SRB assay as previously described [25]. Briefly, exponentially growing cells were collected using 0.25% trypsin-EDTA and plated in 96-well plates at 1000–2000 cells/well. Cells were exposed to test compounds for 72 h and subsequently fixed with TCA (10%) for 1 h at 4 °C. After washing trice, cells were exposed to 0.4% SRB solution for 10 min in dark then washed with 1% glacial acetic acid. After drying overnight, Tris-HCl was used to dissolve SRB-stained cells and color intensity was measured at 540 nm. Doxorubicin^®^ (Sigma Company, Suffolk County, NY, USA) was used as a standard cytotoxic drug.

The dose response curve of each compound was analyzed using Emax model (Equation (1)):

(1) $$\%\;\mathrm{Cell}\;\mathrm{viability}=\left(100-R\right)\times \left(1-\frac{{\left[D\right]}^{m}}{K_{d}{}^{m}+{\left[D\right]}^{m}}\right)+R$$

where [R] is the residual unaffected fraction (the resistance fraction), [D] is the drug concentration used, [Kd] or IC_50_ is the drug concentration that produces a 50% reduction of the maximum inhibition rate and [m] is a Hill-type coefficient. Absolute IC_50_ is defined as the drug concentration required to reduce absorbance by 50% of control (i.e., Kd = absolute IC_50_ when R = 0 and E_max_ =100-R) [26].

### 4.6. Cell Cycle Analysis

To assess the effect of tested compounds on cell cycle distribution, cells were treated with the pre-calculated IC_50_ for each compound for 24 h. After treatment, cells were harvested with trypsin/EDTA, washed twice with ice-cold PBS and then re-suspended in 0.5 mL of PBS. Cells were fixed with 60% ice-cold ethanol for a minimum of one hour at 4 °C and stored at −20 °C. After two washes with PBS, cells were resuspended in 1 mL of PBS containing 50 μg/mL RNAase-A and 10 μg/mL propidium iodide (PI) and incubated for 20 min in dark at room temperature. DNA content was analyzed by ACEA Novocyte™ flowcytometer (ACEA Biosciences Inc., San Diego, CA, USA) and PI fluorescent signals were analyzed using FL2 detector (λex/em 535/617). For each sample, 12,000 events were acquired and cell cycle distribution was calculated using ACEA NovoExpress™ software (ACEA Biosciences Inc., San Diego, CA, USA) [27].

### 4.7. Apoptosis Assessment

To assess the effect of tested compounds on programmed cell death, early/late apoptosis and necrosis cell populations were measured using Annexin V-FITC/PI apoptosis/necrosis detection kit (Abcam Inc., Cambridge Science Park, Cambridge, UK). Briefly, cells under investigation were treated with the pre-determined IC_50_’s for each compound for 24 h, then cells were collected by trypsinization, washed twice with ice-cold PBS, and re-suspended in 0.5 mL of annexin V-FITC/PI solution for 30 min in the dark according to manufacturer protocol. After staining at room temperature, cells were injected through ACEA Novocyte™ flowcytometer (ACEA Biosciences Inc., San Diego, CA, USA) and analyzed for FITC and PI fluorescent signals using FL1 and FL2 signal detector, respectively (λex/em 488/530 nm for FITC and λex/em 535/617 nm for PI). For each sample, 12,000 events were acquired and positive FITC and/or PI cells were quantified by quadrant analysis and calculated using ACEA NovoExpress™ software (ACEA Biosciences Inc., San Diego, CA, USA) [28].

### 4.8. Apoptosis Gene Expression Analysis

Real-time polymerase chain reaction (PCR) was performed to assess the expression of p53 and TP53INP1 genes after treatment with the pre-determined IC_50_’s of cattleianal and cattleianone. After 24h of treatment, RNA was extracted using mirVana™ RNA isolation kit (Invitrogen, Carlsbad, CA, USA). The RNA and purity were confirmed (A260/280 > 2.0) using DeNovix DS-11™ microvolume spectrophotometer (Thermo Fisher Scientific, Wilmington, DE, USA). Subsequently, the total RNA samples of all treatments were reverse transcribed to construct a cDNA library using the SuperScript™ Master Mix kit (Invitrogen, Carlsbad, CA, USA). The cDNAs were then subjected to quantitative real-time PCR reactions using TaqMan^®^ Gene Expression Assay (Applied Biosystems, Foster City, CA, USA). *p53* forward primer was 3′-CCTCACCATCATCACACTGG-5′, and backward primer was 5′-CTGAGTCAGGCCCTTCTGTC-3′; TP53INP1 forward primer was 3′-TTCCTCCAACCAAGAACCAGA-5′, and backward primer was 5′-GCTCAGTAGGTGACTCTTCACT-3′. Housekeeping β-actin gene was used as a reference gene with forward primer 3′-GAGAGGCGGCTAAGGTGTTT-5′ and backward primer 5′-TGGTGTAGACGGGGATGACA-3′; normalized fold changes for all genes of interest were calculated using the formula: 2^−ΔΔCq^.

### 4.9. Assessment of Active Caspase-3 Concentration

To confirm the effect of cattleianal and cattleianone on apoptosis, the active caspase-3 level was measured using Quantikine^®^ caspase-3 ELISA kit (R&D Systems, Inc., Minneapolis, MN, USA). Briefly, after cattleianal and cattleianone treatment for 24 h, cells were collected and washed, then incubated with the biotin-ZVKD-fmk inhibitor for 1 h. Cells were transferred into the wells of a microplate pre-coated with an anti caspase-3 monoclonal antibody and washed to remove any unbound substances. Streptavidin conjugated to horseradish peroxidase was added to the wells and bound to the biotin on the inhibitor. Following a wash to remove any unbound Streptavidin-HRP, a substrate solution was added to the wells. The enzyme reaction yields a blue product that turned yellow when a Stop Solution was added. The optical density of each well was determined within 30 min, using a microplate reader set to 450 nm with a wavelength correction at 540 nm or 570 nm. The concentrations of active caspase-3 were calculated from a standard curve of, constructed with known concentrations of active caspase-3. Caspase concentration was expressed as ng/mg protein. Protein concentrations were determined using Bradford method and purified bovine serum albumin was used as a standard calibration protein.

### 4.10. Statistical Analysis

Data are presented as mean ± SEM using GraphPad prism™ software (GraphPad software Inc., La Jolla, CA, USA) for windows version 5.00. Analysis of variance (ANOVA) with Dunnett’s post hoc test was used for testing the significance using SPSS^®^ for windows, version 17.0.0. *p* < 0.05 was taken as a cut off value for significance.

## 5. Conclusions

Phytochemical investigation of the leaves of *P. cattleianum* resulted in the isolation of two new meroterpenoid phloroglucinols (cattleianal and cattleianone) with selective cytotoxic activity against HT-29, Hep-2, MCF7 and MDA-MB231 cancer cell lines. In colorectal adenocarcinoma cells (HT-29), both compounds showed an anti-proliferative effect by causing G_0_/G_1_ cell cycle arrest which was accompanied by induction of early and late apoptosis. Meanwhile, HCT-116 cells, the most resistant to both compounds, underwent only necrosis after 24 h of incubation implicating non-specific cell death. The study emphasizes previous work regarding the potential use of phloroglucinol derivatives as selective anti-proliferative/cytotoxic agents and encourages further research in this group of compounds as a scaffold for development of selective chemotherapeutic agents.

## Figures and Tables

**Table 1:** Results of the cytotoxic effect of cattleianal and cattleianone on different human cell lines.

Compound	HFB4	HepG2	HEp2	MCF7	HCT-116	HT-29	MDA-MB-231
Cattleianal (1)	>100	40.3 ± 1.35	28.3 ± 0.1	44.8 ± 2.2	>100	35.2 ± 4.2	64.4 ± 5.6
Cattleianone (2)	>100	22.0 ± 0.9	33.6 ± 2.8	23.7 ± 1.6	70.5 ± 1.6	32.1 ± 1.4	38.7 ± 3.5
Doxorubicin	7.36 ± 0.33	7.73 ± 0.8	8.0 ± 0.9	7.73 ± 0.6	0.76 ± 0.1	0.78 ± 0.1	0.83 ± 0.2

IC_50_’s are expressed in μM ± SEM.

## Data Availability

The data presented in this study are available in the Supplementary Materials.

## Supplementary Materials

Figure S1: EI-MS spectrum of compound **1**. Figure S2: ^1^H-NMR spectrum of compound **1**. Figure S3: ^13^C-NMR spectrum of compound **1**. Figure S4: HMBC spectrum of compound **1**. Figure S5: Expanded HMBC spectrum of compound **1**, Figure S6: HSQC spectrum of compound **1**. Figure S7: expanded HSQC spectrum of compound **1**. Figure S8: HRMS (Negative Mode) of compound 2. Figure S9: UV spectrum of compound **2** (λ_max_ = 294 and 341 nm), recorded in methanol. Figure S10:^1^H-NMR spectrum of compound **2**. Figure S11: ^13^C-NMR spectrum of compound **2**. Figure S12: HMBC spectrum of compound **2**. Figure S13: Expanded HMBC spectrum of compound **2**. Figure S14: HSQC spectrum of compound **2**. Figure S15: Expanded HSQC spectrum of compound **2**, Figure S16: H-H COSY spectrum of compound **2**, Figure S17: Expanded aliphatic region of H-H COSY spectrum of compound **2**.

## Sample Availability

Not available.
