# Supplementary material for: Cattleianal and Cattleianone: Two New Meroterpenoids from Psidium cattleianum Leaves and Their Selective Antiproliferative Action against Human Carcinoma Cells
Source: Molecules. 2021 May 13;26(10):2891. doi: 10.3390/molecules26102891 (PMC8153265; doi:10.3390/molecules26102891)

Electronic Supplementary material

Cattleianal and Cattleianone: Two new meroterpenoids from Psidium cattleianum leaves and their selective antiproliferative action against human carcinoma cells

Engy A. Mahrous[1], Ahmed M. Al-Abd [2, 3]*, Maha M. Salama [1,4], Magda M. Fathy [1], Fathy M. Soliman[1] and Fatema R. Saber[1]

[1] Pharmacognosy Department, Faculty of Pharmacy, Cairo University, Kasr el-Aini street, Cairo, 11562, Egypt.

[2] Department of Pharmaceutical Sciences, College of Pharmacy & Thumbay Research Institute of Precision Medicine, Gulf Medical University, Ajman, UAE.

[3] Pharmacology Department, Medical Division, National Research Centre, Cairo, Egypt.

[4] Pharmacognosy Department, Faculty of Pharmacy, The British University in Egypt, El-Sherouk City,11837, Cairo, Egypt.

Correspondence: Ahmed M. Al-Abd*, ahmedmalabd@pharma.asu.edu.eg, Tel. No: +971 56 464 2929.

Figure S1: Electron impact (EI) mass spectrum of compound 1

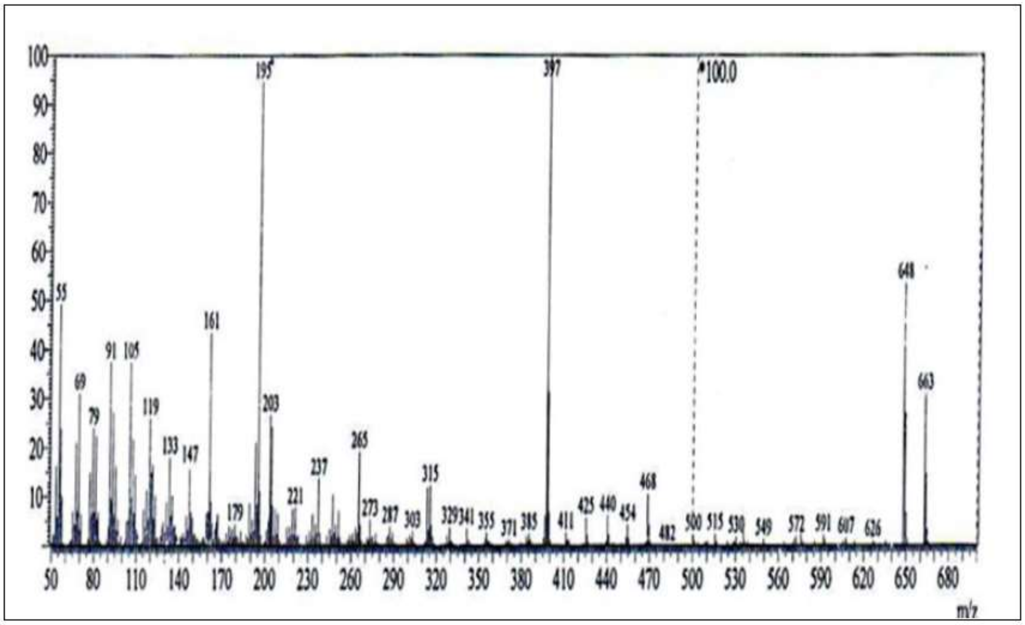

Figure S2: ^{1}H-NMR spectrum of compound 1

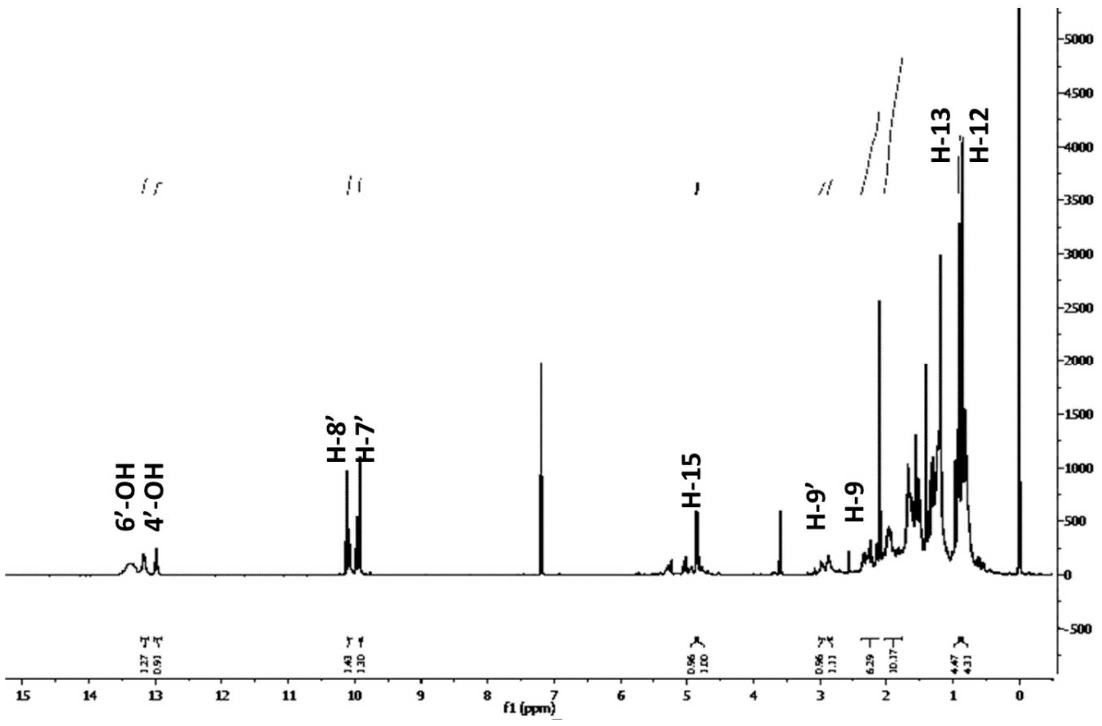

Figure S3:^{13}C-NMR spectrum of compound 1

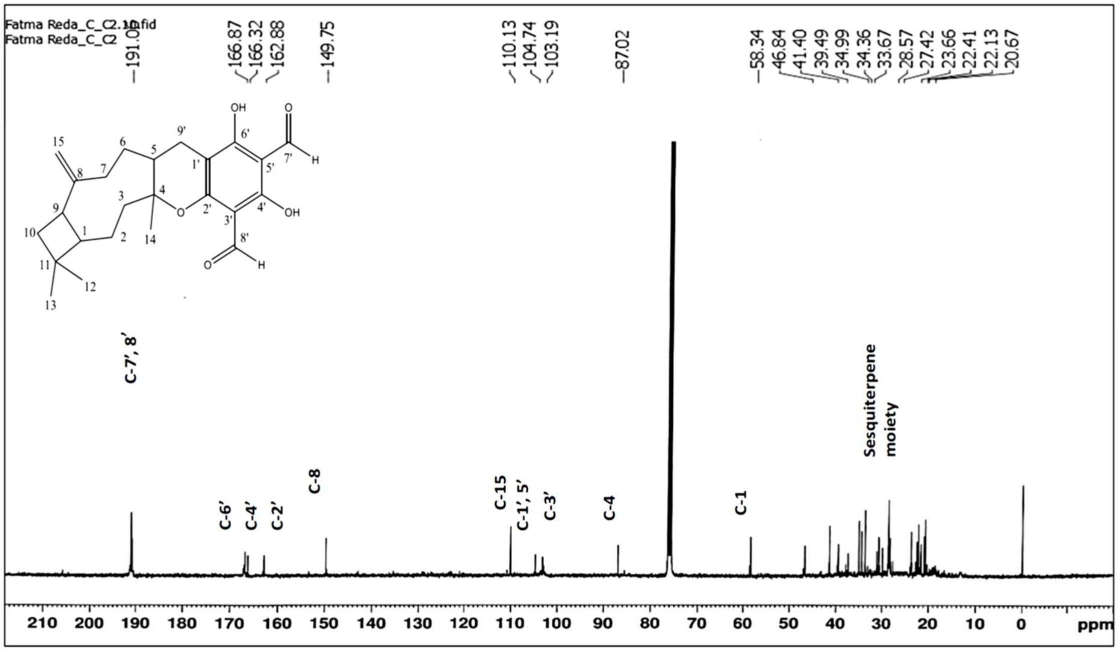

Figure S4:^{1}H-^{13}C HMBC spectrum of compound 1

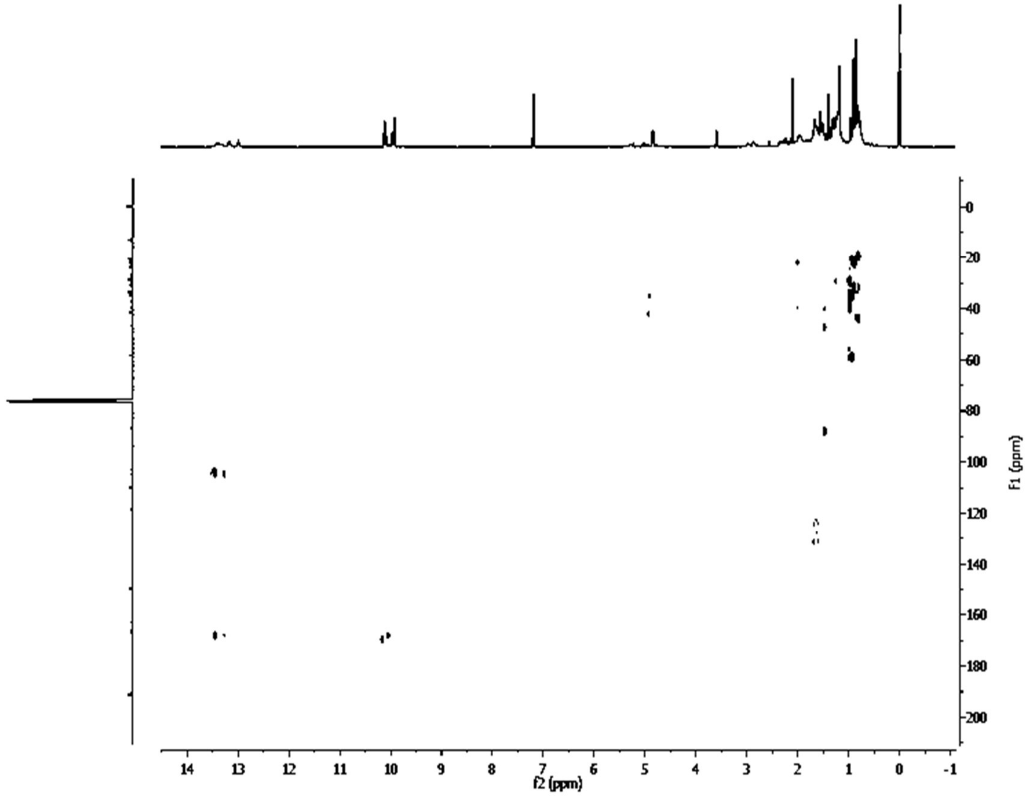

Figure S5: Expansion of the aliphatic region of the HMBC spectrum of compound 1

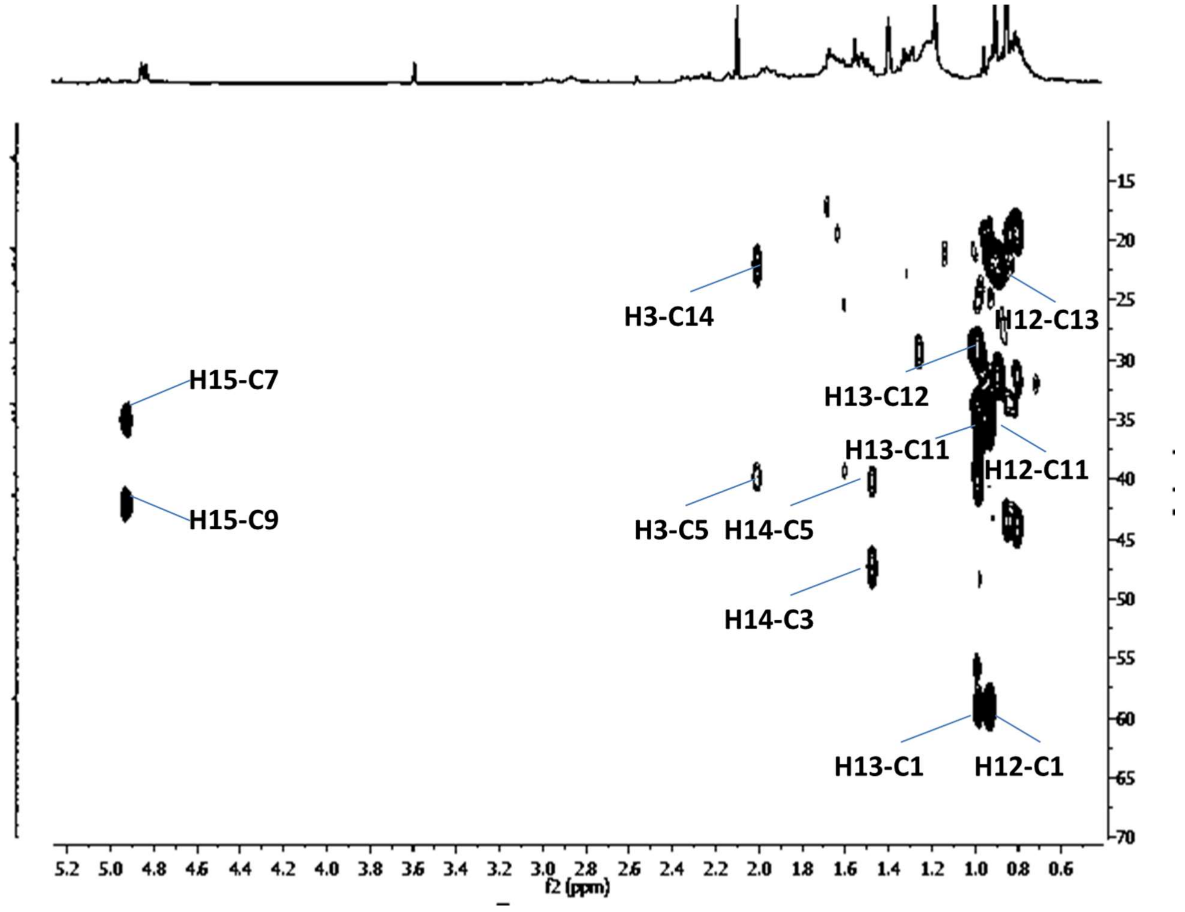

Partially expanded HMBC spectrum of compound 1 showing the long range H-C coupling in the aliphatic region δ_H 0.5-5.3, δ_C 10-70

Figure S6: 1H-13C HSQC spectrum of compound C1

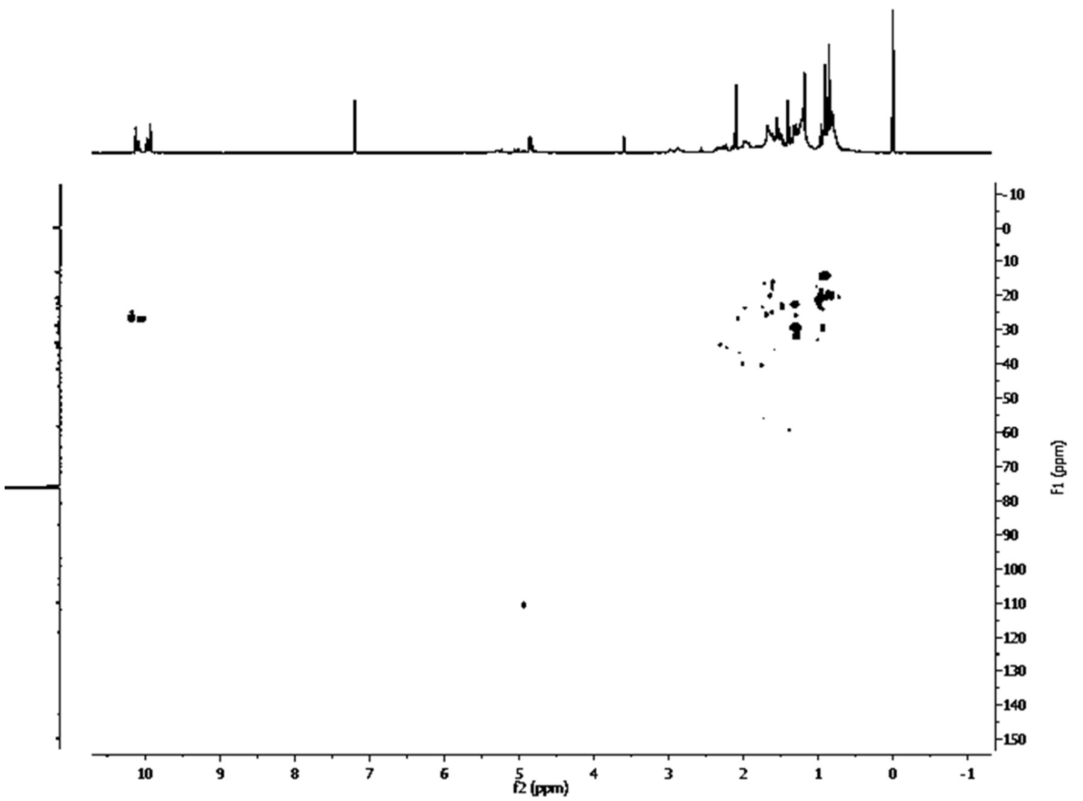

Figure S7: Expansion of the aliphatic region of HSQC spectrum of compound 1

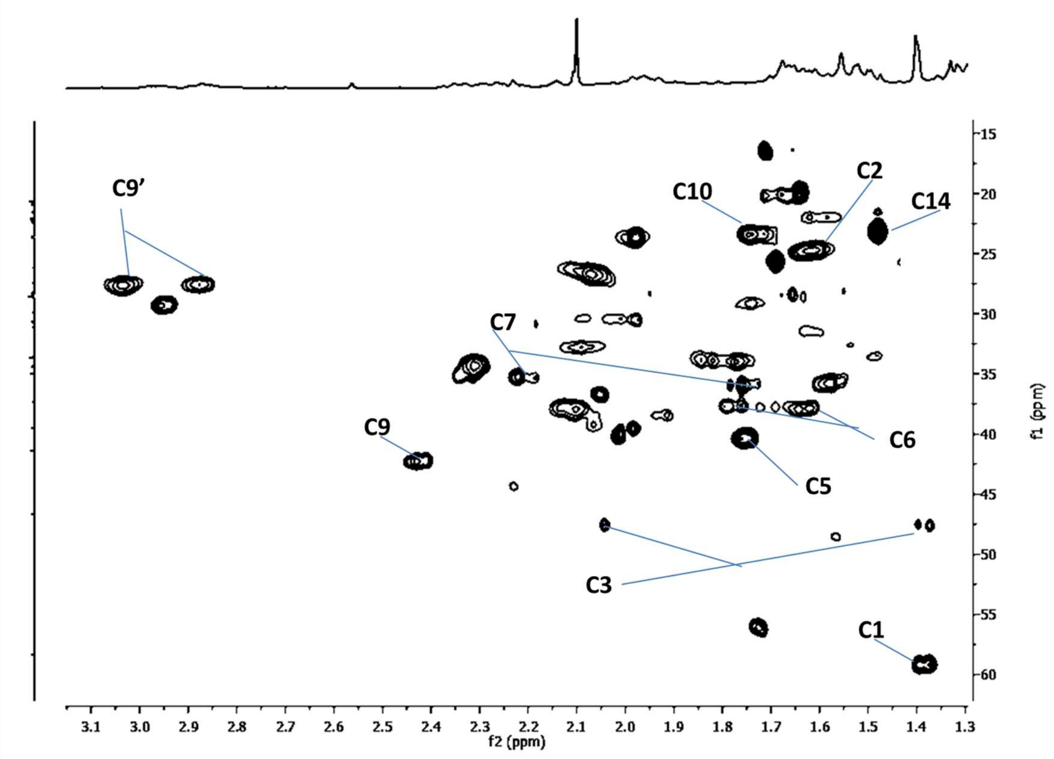

Partially expanded HSQC spectrum of compound 1 showing the long range H-C coupling in the aliphatic region δ_H 1.3-3.2, δ_C 14-65

Figure S8: High Resolution electrospray ionization (ESI-MS) spectrum of compound 2

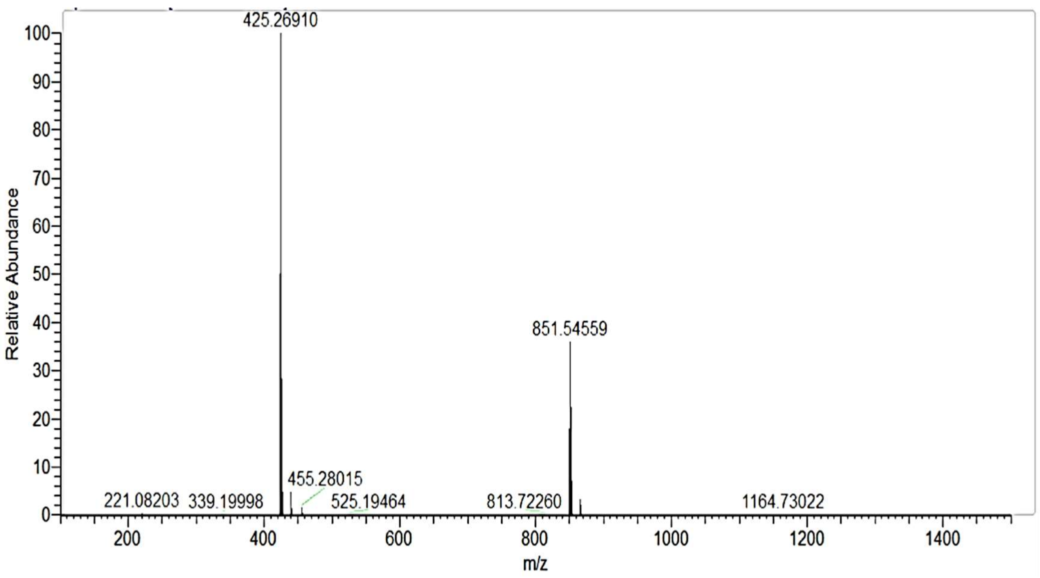

Figure S9: UV spectrum of compound 2

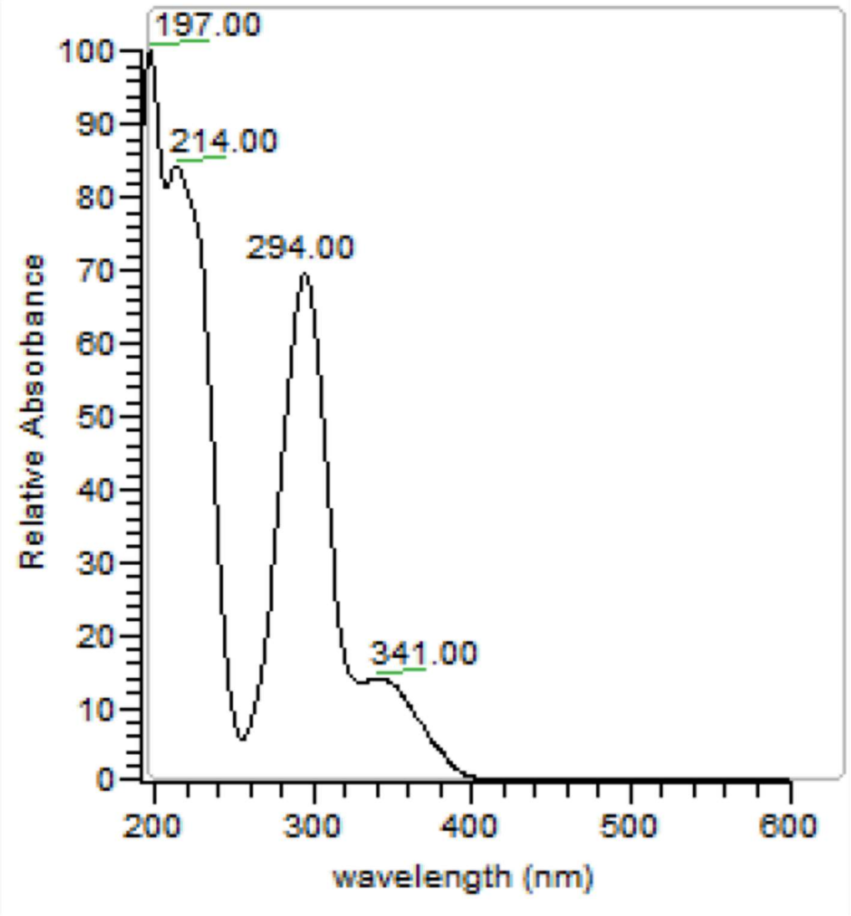

Figure S10: ^{1}H-NMR spectrum of compound 2

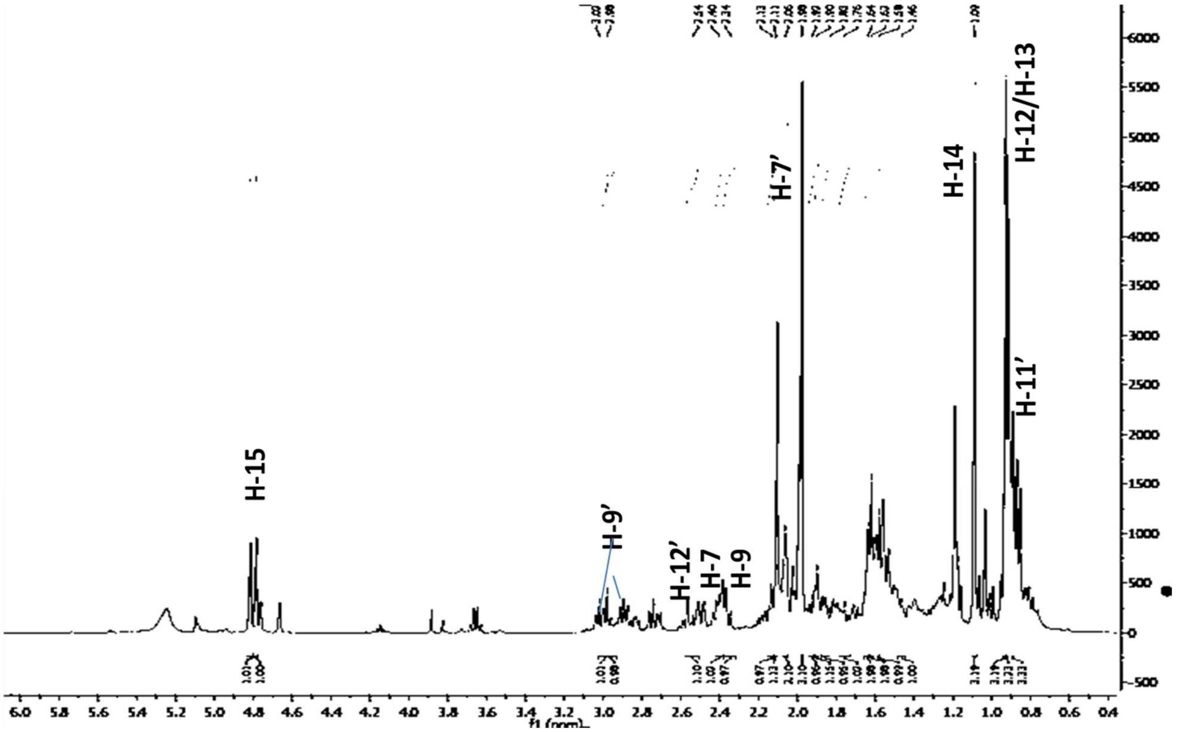

Figure S11: ^{13}C- NMR spectrum of compound 2

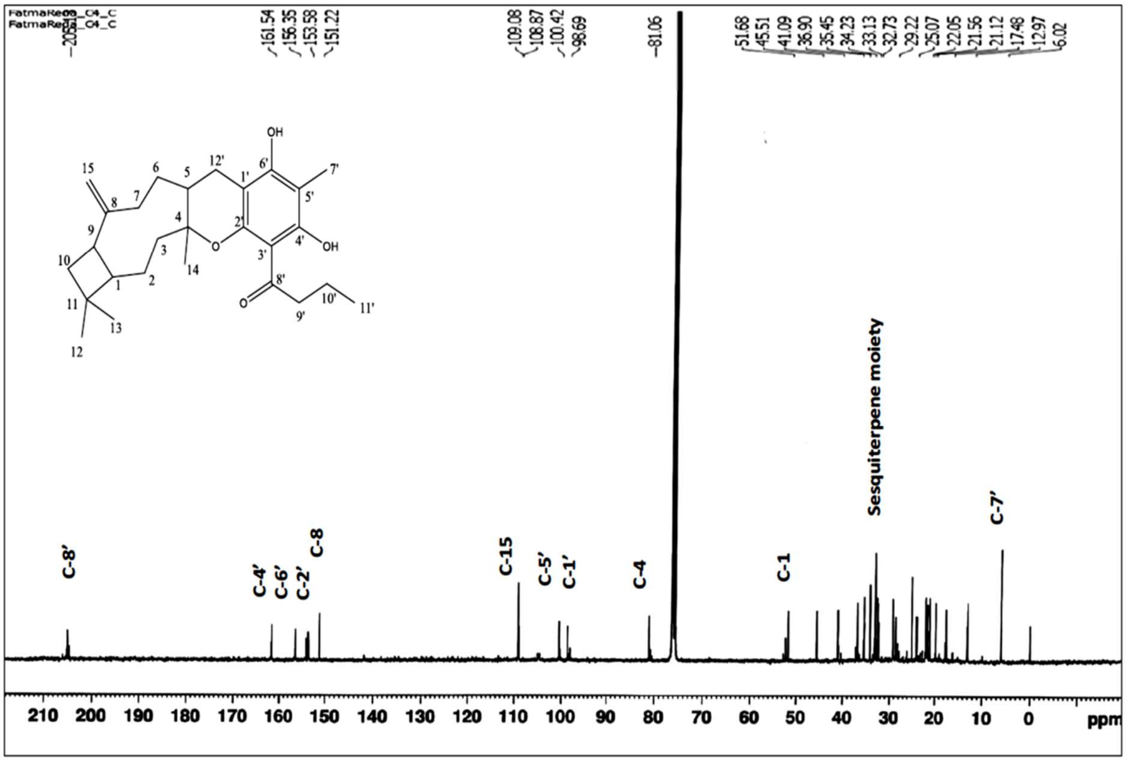

Figure S12: HMBC NMR spectrum of compound 2

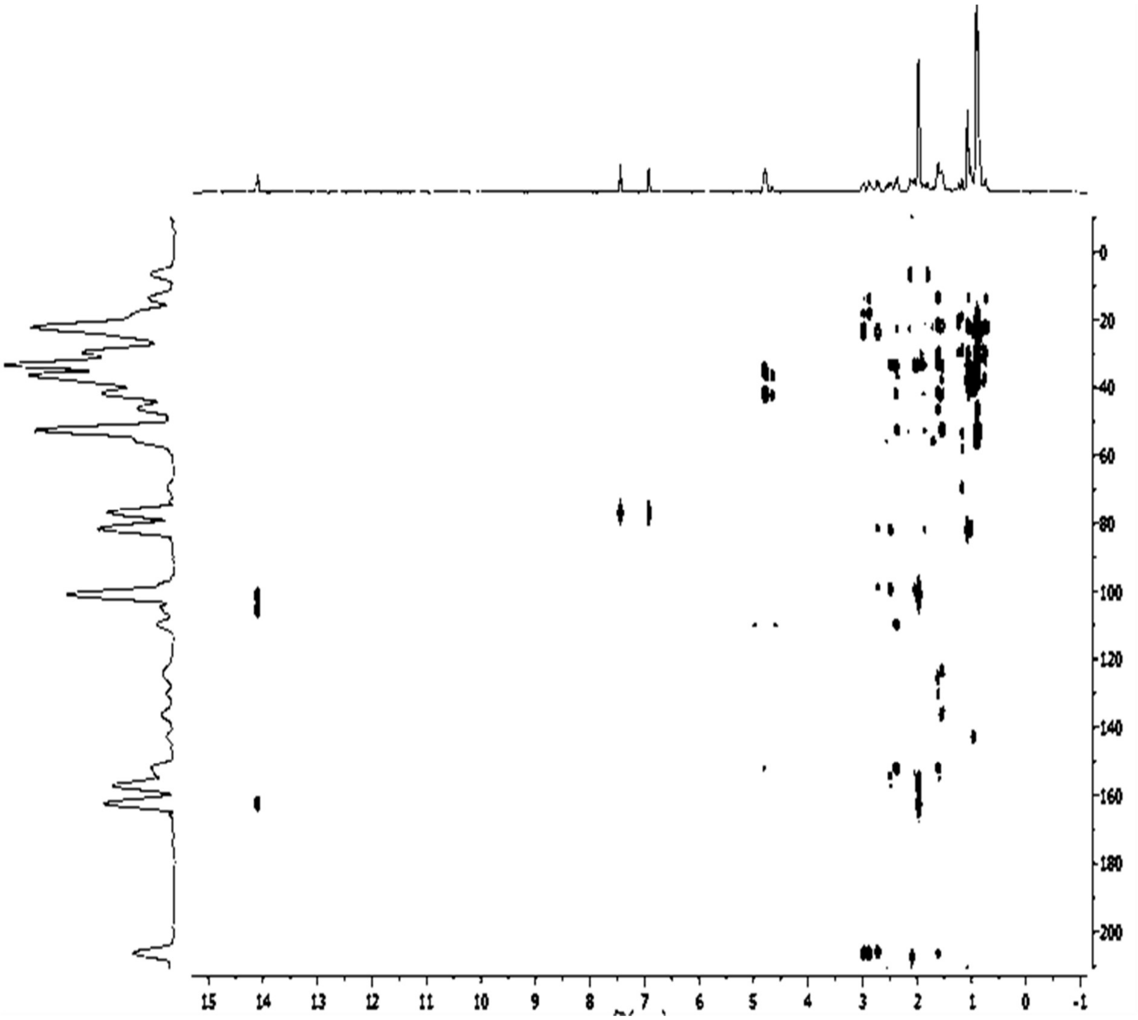

Figure S13: Expansion of the HMBC spectrum of the aliphatic region of compound 2

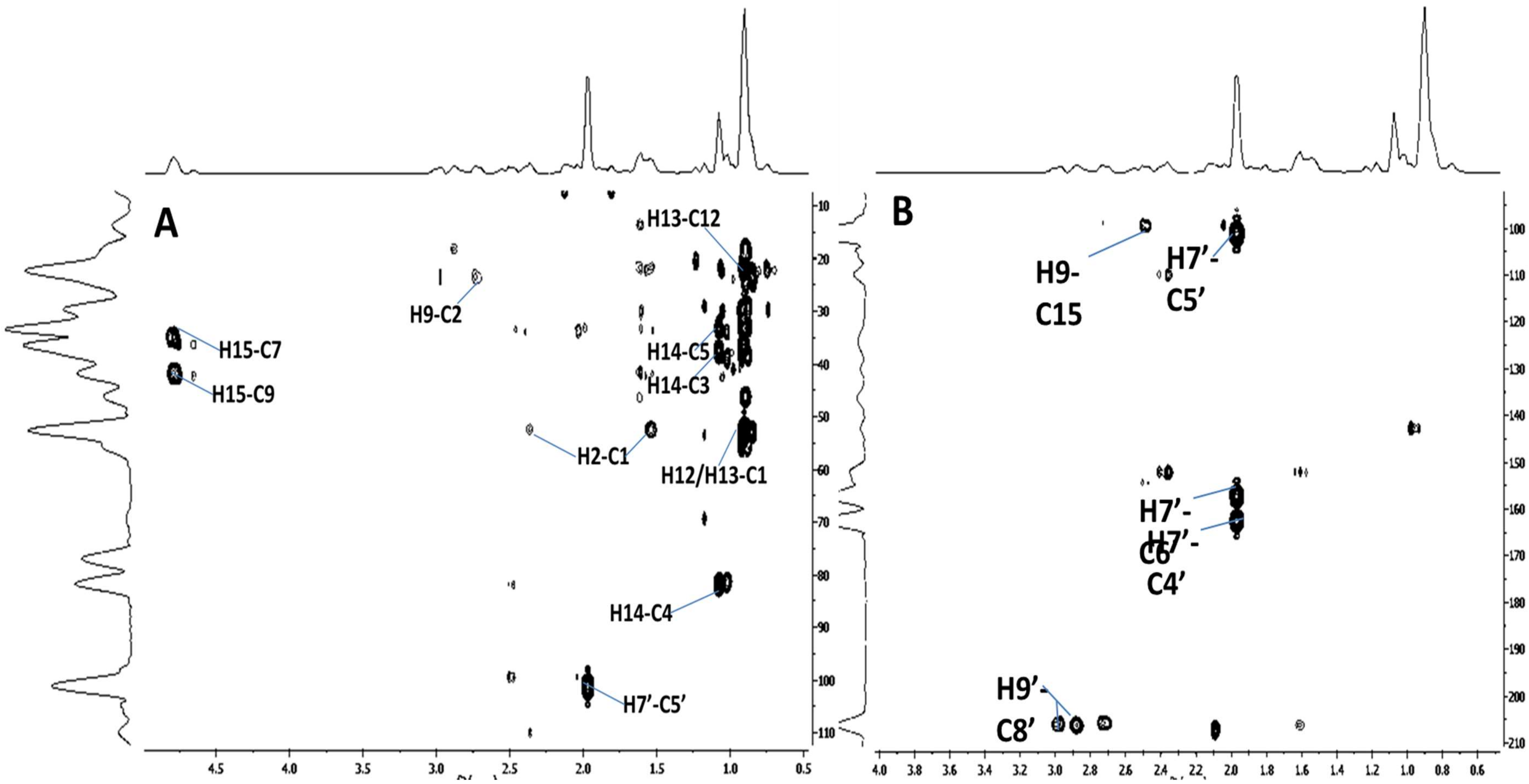

Partially expanded HMBC spectrum of compound 2 showing the long range H-C coupling in the aliphatic region A) in the region δ_H 0.5-5.0, δ_C 10-110 and B) in the region δ_H 0.5-4.0, δ_C 100-210

Figure S14: HSQC spectrum of compound 2

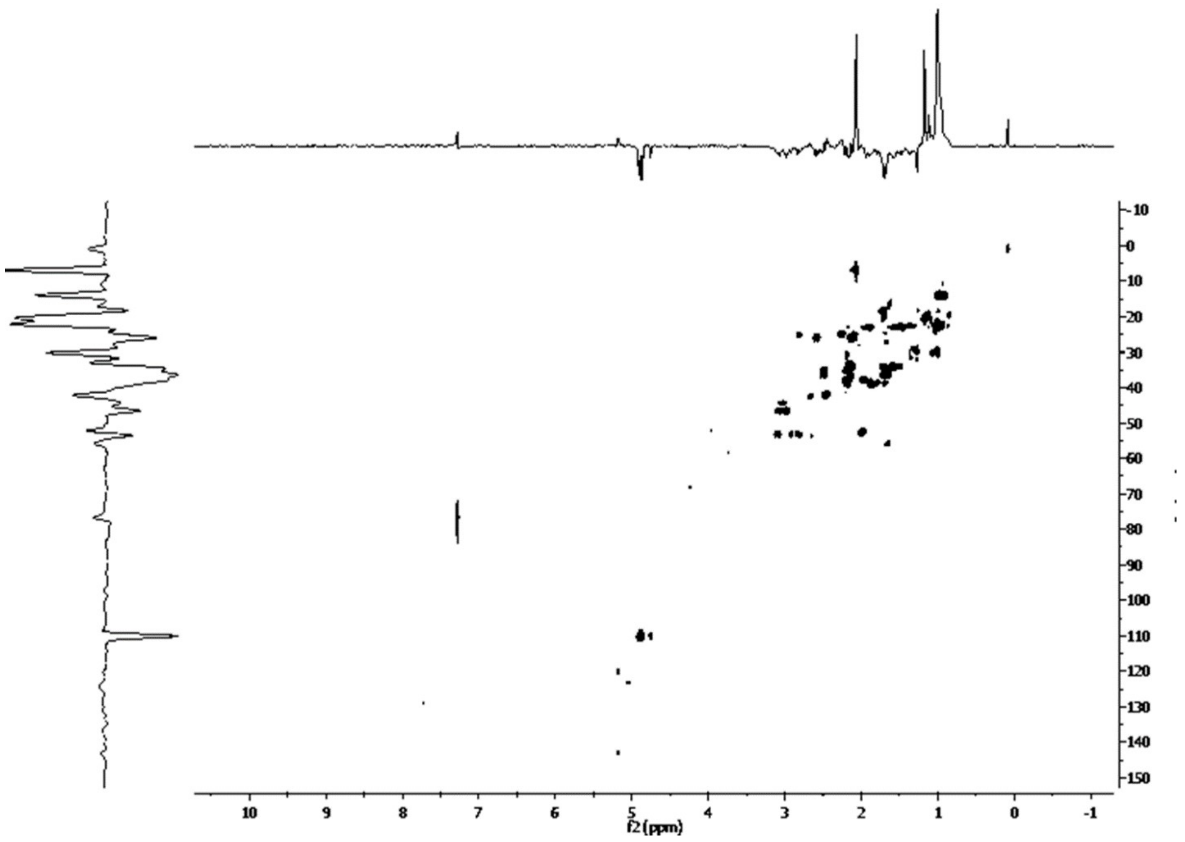

Fig S15: Expansion of HSQC spectrum of compound 2 at the aliphatic region

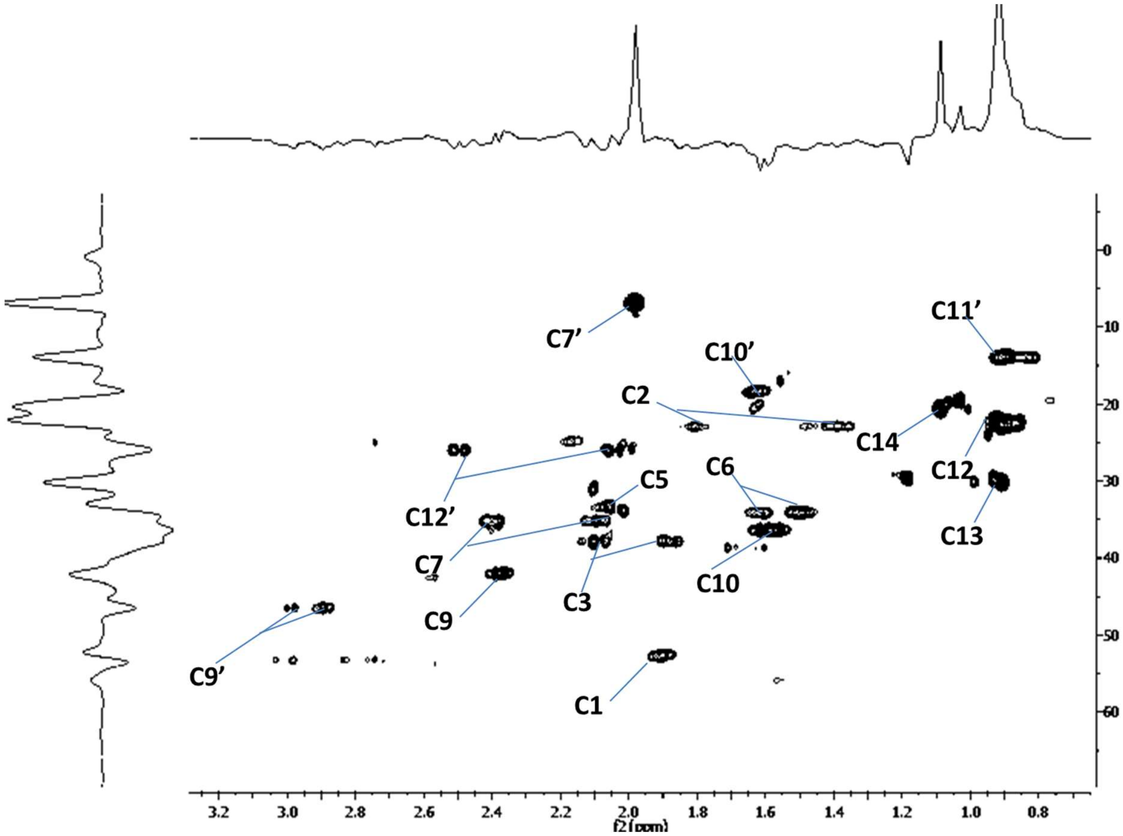

Partially expanded HSQC spectrum of compound 2 showing the long range H-C coupling in the aliphatic region δ_H 0.5-3.4, δ_C 0-70

Fig S16: H-H COSY spectrum of compound 2

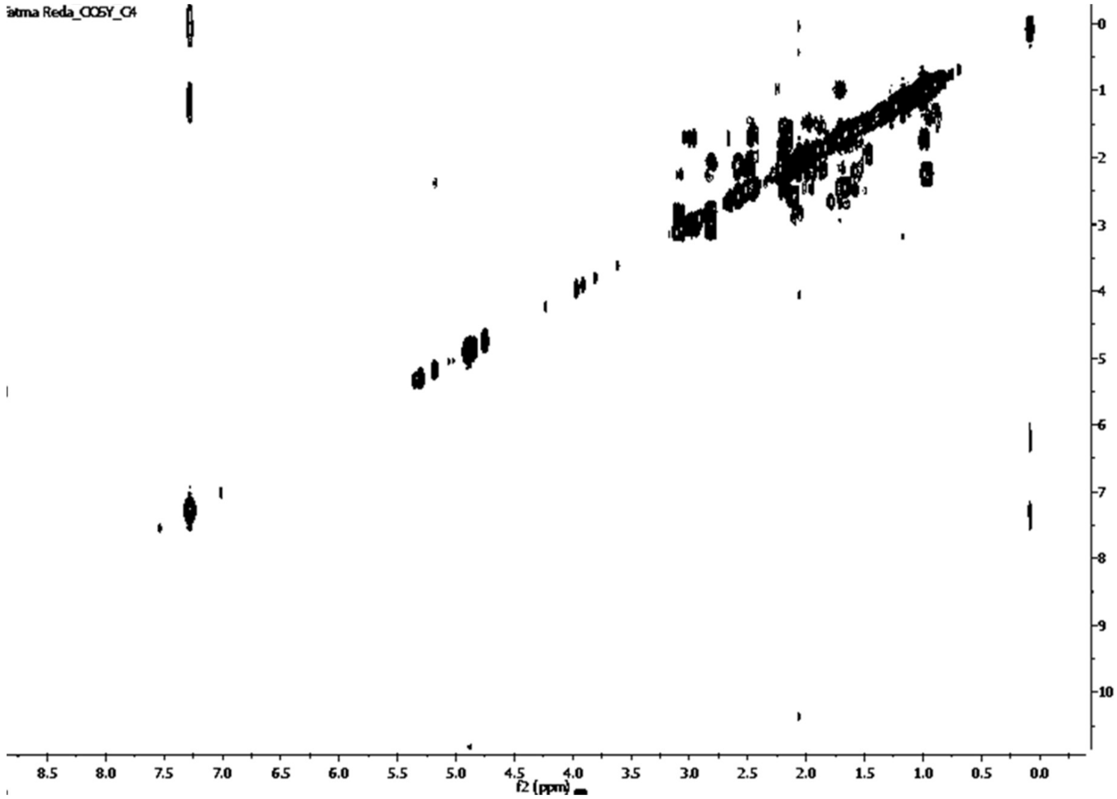

Fig S17: Expanded aliphatic region of H-H COSY spectrum of compound 2

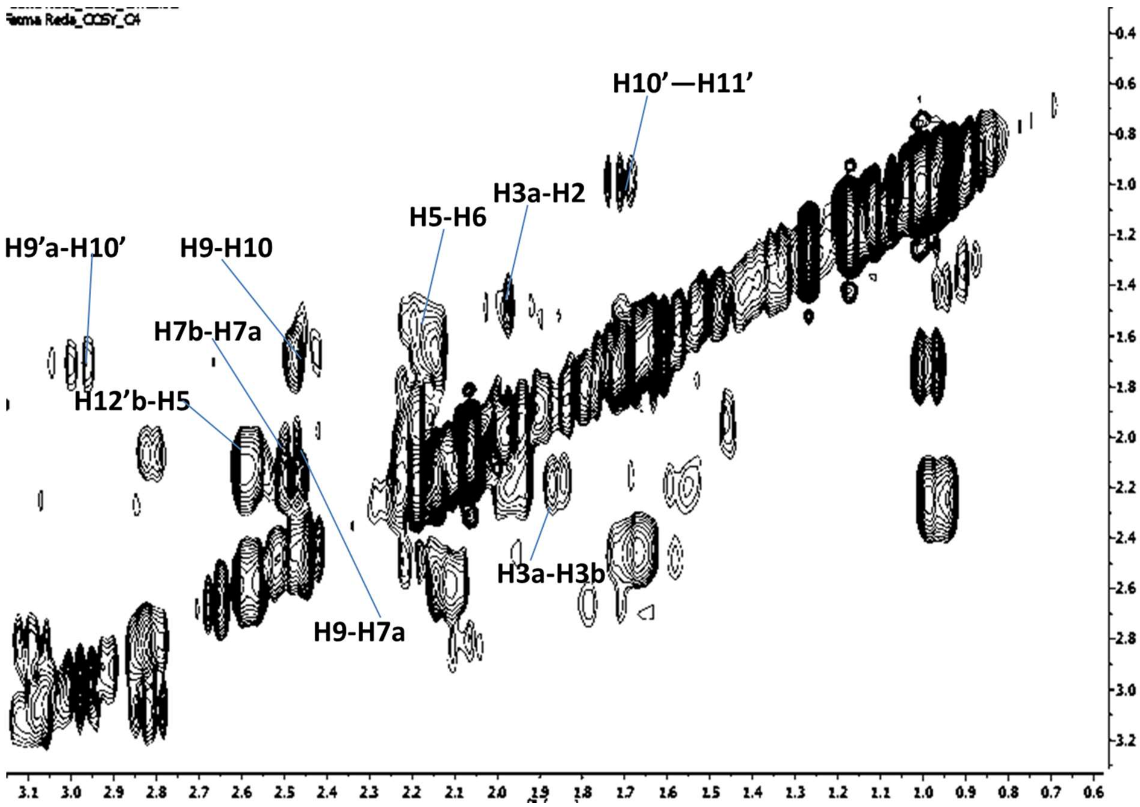

Supplement: Supplementary file 1 [file molecules-26-02891-s001.zip › molecules-1202228-supplementary.pdf]
